# Supplementary material for: Active oxygen species mediate the iron-promoting electrocatalysis of oxygen evolution reaction on metal oxyhydroxides
Source: Nat Commun. 2023 Oct 26;14:6826. doi: 10.1038/s41467-023-42646-z (PMC10603066; doi:10.1038/s41467-023-42646-z)
Supplement: Supplementary file 1 — Supplementary Information [file 41467_2023_42646_MOESM1_ESM.pdf]

Supplementary Information for Active Oxygen Species Mediate the Iron-Promoting Electrocatalysis of Oxygen Evolution Reaction on Metal Oxyhydroxides

Qu Jiang,[1] Sihong Wang,[1] Chaoran Zhang,[1] Ziyang Sheng,[1] Haoyue Zhang,[1] Ruohan Feng,[1] Yuanman Ni,[1] Xiaoan Tang,[1] Yichuan Gu,[1] Xinhong Zhou,[1] Seunghwa Lee,[2] Di Zhang,[1] Fang Song[1,*]

[1]State Key Laboratory of Metal Matrix Composites, School of Materials Science and Engineering, Shanghai Jiao Tong University, Shanghai 200240, China

[2]Department of Chemical Engineering, Changwon National University, Changwon-Si, Gyeongsangnam-do 51140, South Korea

[*] Corresponding authors: songfang@sjtu.edu.cn

1. Supplementary Methods

Reagents and Materials

Commercial Fe, Co, Ni, Cu, Ag, Au, Ti, Nb, and Sn foils were purchased from ZhongNuo Advanced Material (Beijing) Technology Co., Ltd. All reagents were analytical grade without further purification. Ferric nitrate nonahydrate ($Fe(NO_3)_3 \cdot 9H_2O$) and potassium hydroxide (KOH) were purchased from Shanghai Macklin Biochemical Co., Ltd. Nickel nitrate hexahydrate ($Ni(NO_3)_2 \cdot 6H_2O$) was purchased from Shanghai Aladdin Biochemical Technology Co., Ltd. Hydrochloric acid (HCl), ethanol and acetone were purchased from Sinopharm Chemical Reagent Co., Ltd. The water used throughout all experiments was dual distilled water.

Materials characterizations

Scanning electron microscope (SEM) images were taken using JSM-7800F operating at 5 keV. X-ray photoelectron spectroscopy (XPS) was collected on an AXIS Ultra DLD using a monochromated Al-Kα X-ray source (hν = 1486.6 eV) operated at 15 kV and 14 mA and a spot size of 300 μm. The crystal structure of foils was characterized by X-ray diffraction (XRD) (Mini Flex 600) using Cu Kα radiation (λ = 1.5418 Å). The iron loading was determined by inductively coupled plasma mass spectrometry (ICP-MS) (NexION2000).

2. Supplementary Figures

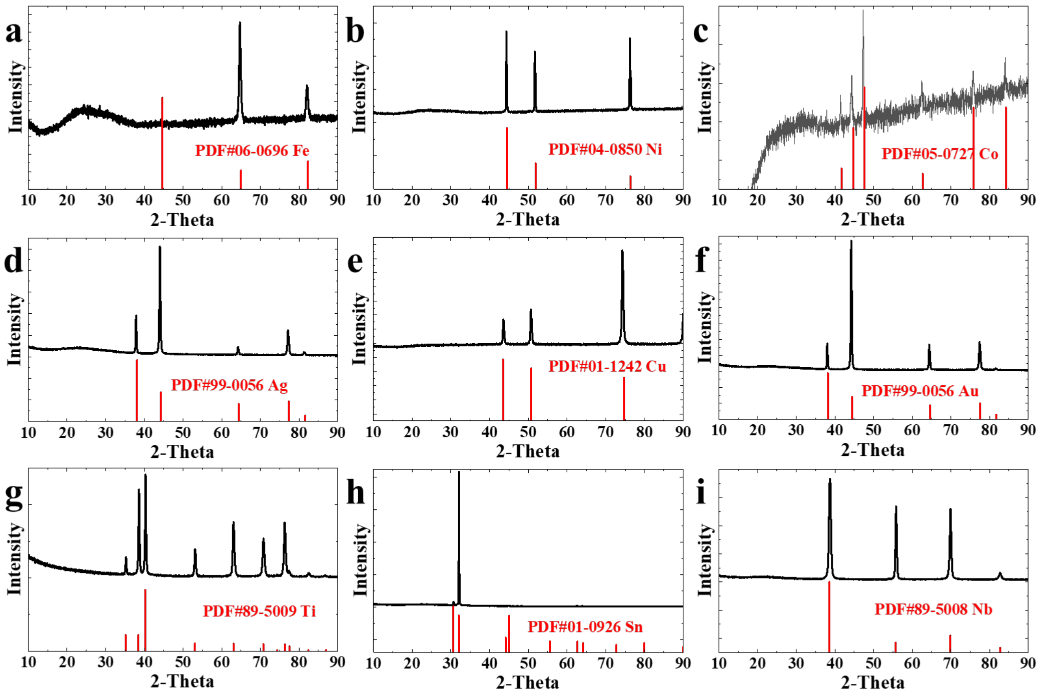

Supplementary Figure 1. XRD patterns of commercial metal foils. (a-i): Fe, Ni, Co, Ag, Cu, Au, Ti, Sn, Nb. Source data are provided as a Source Data file.

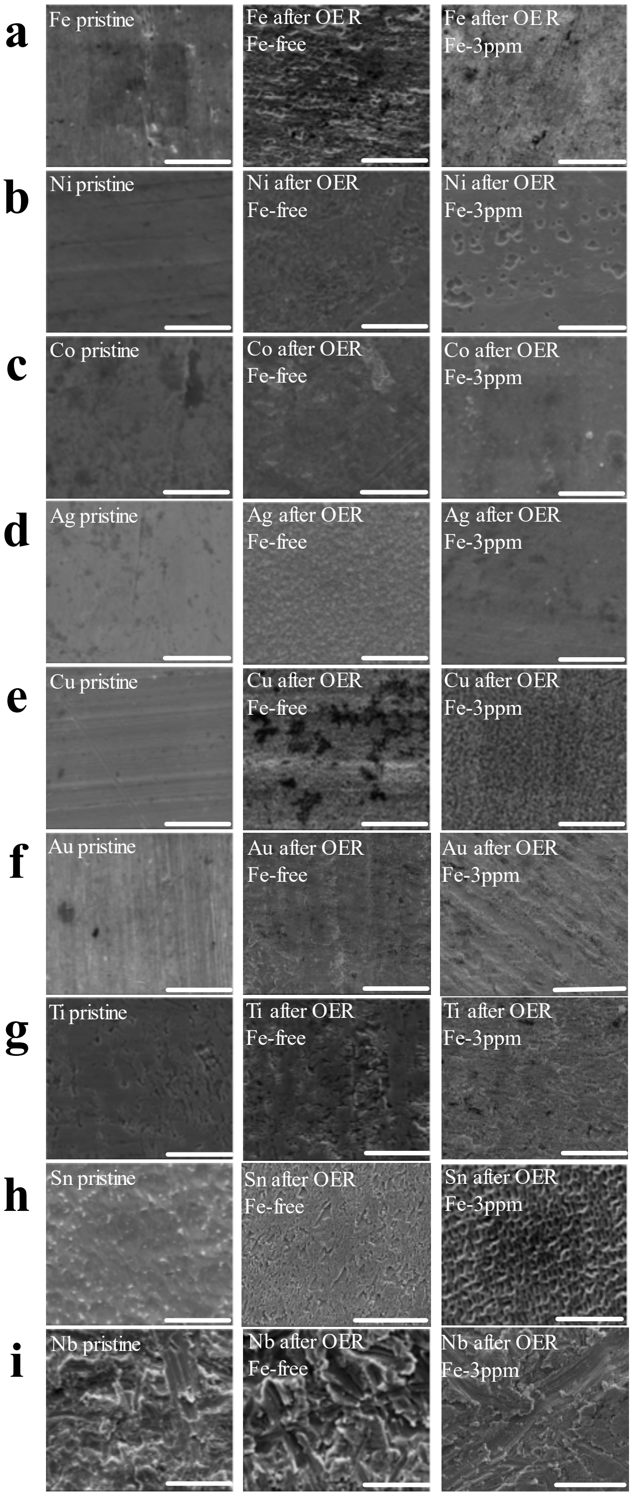

Supplementary Figure 2. SEM images of metal foils under different conditions.

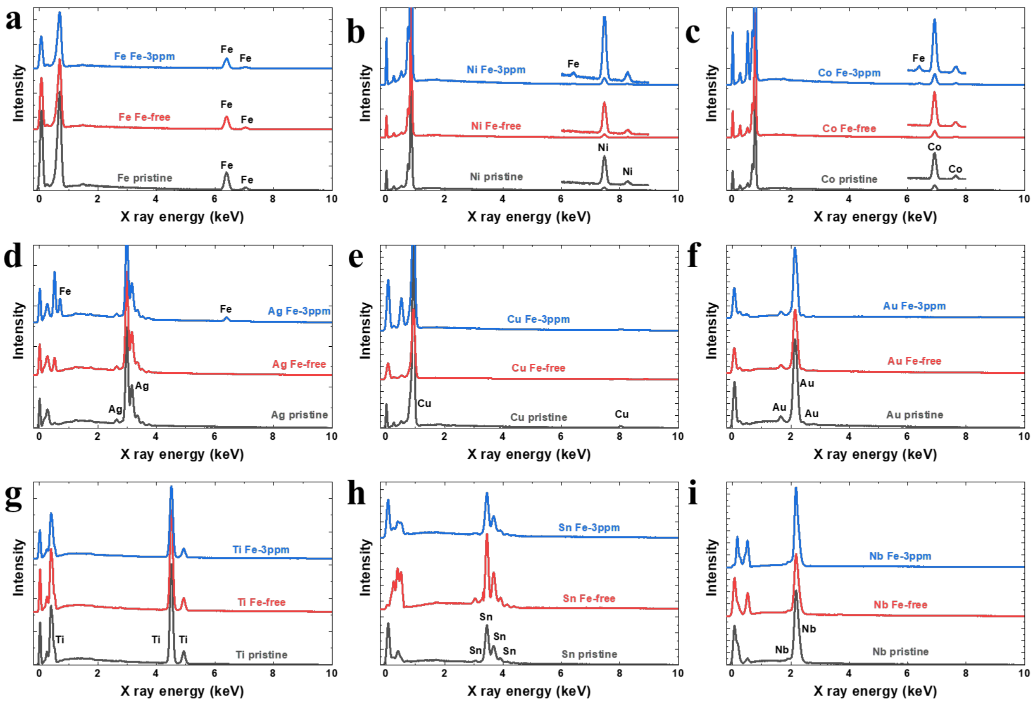

Supplementary Figure 3. EDS spectrum of metal foils under different conditions. Source data are provided as a Source Data file.

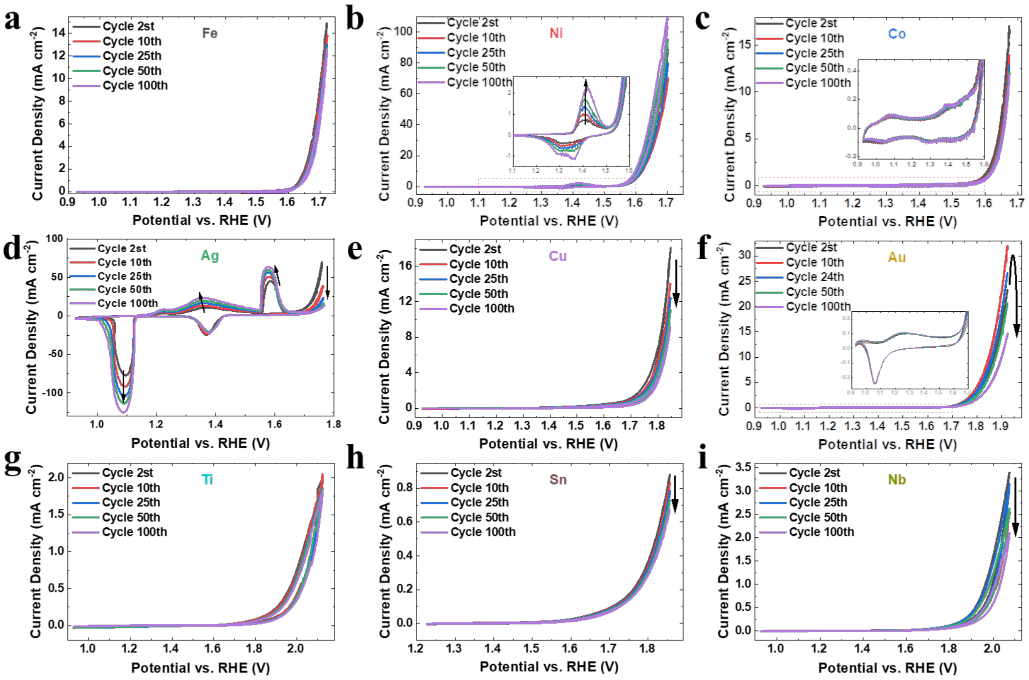

Supplementary Figure 4. 100 repetitive CV curves of metal foils in 1 M Fe-free KOH at a scan rate of 20 mV s^{-1}. (a-i): Fe, Ni, Co, Ag, Cu, Au, Ti, Sn, Nb. Source data are provided as a Source Data file.

Fe: The redox of Fe foil has been extensively studied by Prof. Michael E.G. Lyons and others[1,2]. The redox peaks are located in the potential window of -0.3-0.72 V vs. RHE, which is negative to the potential window here. Therefore, no redox peak was observed in our CV scans (Supplementary Fig. 4a). According to previous electrochemical analysis, FeOOH and FeO_x (including Fe_2O_3 and Fe_3O_4) were produced by the anodic oxidation[1,2]. Source data are provided as a Source Data file.

Ni: Ni electrode exhibits apparent redox peaks in the cyclic voltammetry scans, giving direct evidence for the formation of hydroxides. The redox chemistry of Ni has been extensively studied[1,3,4], where the redox couples are assigned to the transition between metal hydroxides ($Ni(OH)_2$) to oxyhydroxides (NiOOH).

Co: Co electrode also exhibits apparent redox peaks in the cyclic voltammetry scans, giving direct evidence for the formation of hydroxides like Ni electrode. The redox couples of Co are assigned to the transition between metal hydroxides ($Co(OH)_2$) to oxyhydroxides (CoOOH)[1].

Ag: Ag electrode showed a relatively complex CV scan, featuring two redox couples in the potential window. The first redox couple corresponds to the Ag(0)⇋Ag(I) transition, and the second one is attributed to the Ag(I)⇋Ag(II) transition[5]. The enlarging redox peaks and the reducing current density after the OER onset potential indicate the dissolution of Ag in the CV scans (in particular in the beginning ones). To reduce the contribution of corrosion current, the catalytic activities were recorded until the CV scans (including redox peaks) reached a relatively stable state (corresponding to a stable surface).

Cu: The redox peaks of Cu are located in the potential window of 0-1.0 V vs. RHE[6,7], which is out of the potential window here. Therefore, there is no redox peak in the CV scan. However, we do see an apparent oxidizing current at the first CV scan, corresponding to the surface oxidation.

Au: Au electrode manifests a redox couple in the CV scan (Supplementary Fig. 4f). According to the previous report, the anodic oxidization resulted in the formation of monolayer oxides/oxyhydroxides on the surface[8,9].

Ti, Nb, and Sn: The Ti, Nb, and Sn electrodes show no redox peaks in the potential window. This could be ascribed to the formation of a passive layer of metal oxides/hydroxides on the surface. Here we take the Ti electrode as an example to interpret the formation of surface oxides/hydroxides. The anodic behavior of Ti in KOH has been investigated by Prof. Branko N. Popov in 2002[10]. They showed the formation of $Ti(OH)_3$ and $TiO_2 \cdot H_2O$ on the surface. XPS was conducted to confirm the formation of surface oxides and hydroxides. It showed apparent lattice O and surface OH in the O *1s* spectra and cation peaks in the metal spectra (Supplementary Fig. 15). Similar passivation could occur on the surface of Nb and Sn electrodes[11,12], given the absence of redox peaks at the potential window here.

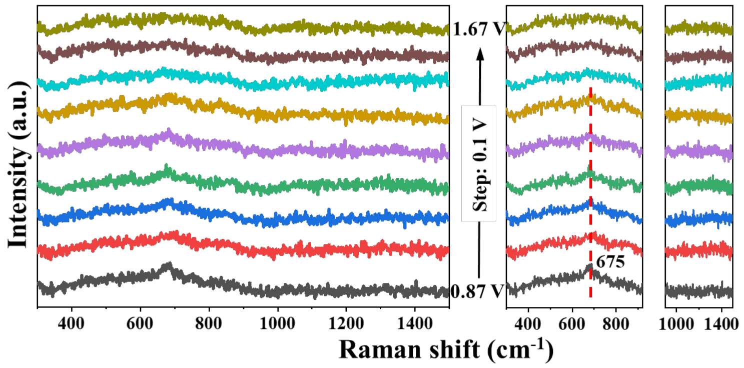

Supplementary Figure 5. In situ Raman spectra of Fe foil in 0.1 M Fe-free KOH under applied potentials ranging from 0.87 V to 1.67 V vs RHE. In situ Raman analysis showed a broad band centered at 675 cm^{-1}. It is difficult to assign the Raman bands owing to the many possible oxides, hydroxides, and oxyhydroxides phases of iron. Based on Prof. A. T. Bell's assignment[3] and spectroscopic analysis including optical reflectance and X-ray absorption spectroscopy[2,13], we suggest the formation of Fe(III) phases of γ-FeOOH (660 cm^{-1}) and γ-Fe_2O_3 (650-740 cm^{-1}). The weak intensity should be ascribed to the low crystallinity. Source data are provided as a Source Data file.

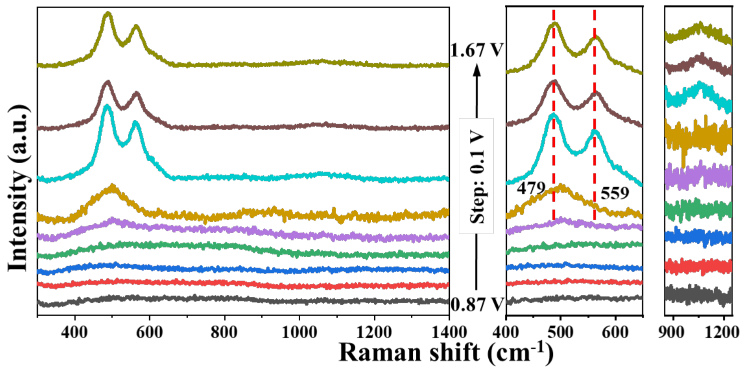

Supplementary Figure 6. In situ Raman spectra of Ni foil in 0.1 M Fe-free KOH under applied potentials ranging from 0.87 V to 1.67 V vs RHE. In situ Raman analysis validated the transition from $Ni(OH)_2$ to Ni(O)OH, showing characteristic peaks of (479 cm^{-1} and 559 cm^{-1} for Ni(O)OH once being electrochemically oxidized over the oxidizing peak potential[3,4]. Source data are provided as a Source Data file.

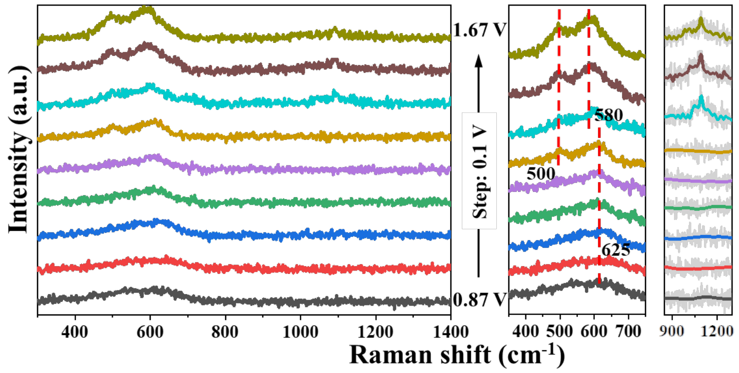

Supplementary Figure 7. In situ Raman spectra of Co foil in 0.1 M Fe-free KOH under applied potentials ranging from 0.87 V to 1.67 V vs RHE. In situ Raman analysis validated the transition from $Co(OH)_2$ to Co(O)OH, showing characteristic peaks of (500 cm^{-1} and 560-580 cm^{-1} for CoOOH once being electrochemically oxidized over the oxidizing peak potential[14,15]. Source data are provided as a Source Data file.

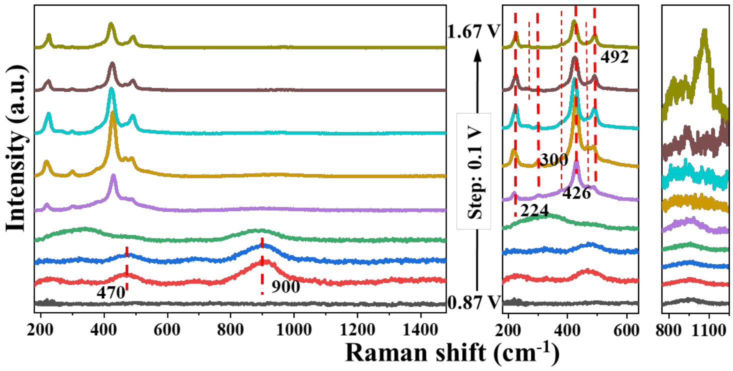

Supplementary Figure 8. In situ Raman spectra of Ag foil in 0.1 M Fe-free KOH under applied potentials ranging from 0.87 V to 1.67 V vs RHE. To get a high-quality signal of active oxygen species, the spectrums in the spectrum range of 150-1500 cm^{-1} were recorded by immediately decreasing the potential to 0 V vs. RHE, where the metallic Ag nanoparticles can magnify the signal via surface enhancement of Raman scattering. In situ Raman analysis showed that electrochemical oxidization led to the formation of Ag_2O (characteristic peaks: 224 cm^{-1}, 300 cm^{-1}, 375 cm^{-1}) and AgO (426 cm^{-1} and 492 cm^{-1})[5,16]. Apparent peaks centered at 470 cm^{-1} and 900 cm^{-1} are attributed to an Ag-O stretching and an Ag-OH binding vibration of OH(ad), indicating the concurrent formation of $Ag(OH)_x$ groups[5,17]. Source data are provided as a Source Data file.

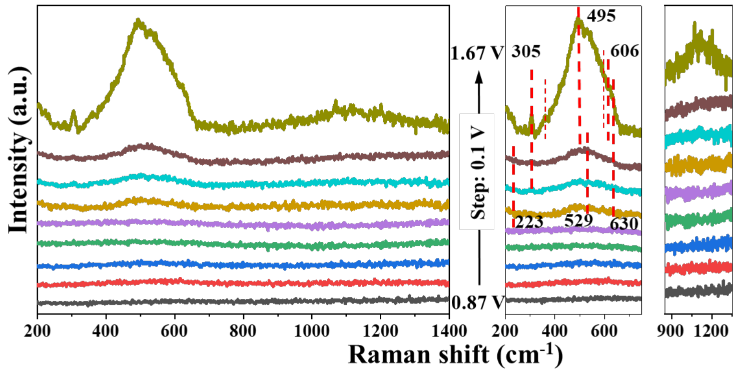

Supplementary Figure 9. In situ Raman spectra of Cu foil in 0.1 M Fe-free KOH under applied potentials ranging from 0.87 V to 1.67 V vs RHE. In situ Raman analysis showed the increasing production of Cu_2O (characteristic peaks: 223 cm^{-1}, 529 cm^{-1}, 630 cm^{-1}), CuO (305 cm^{-1}, 360 cm^{-1}, 591 cm^{-1}), $Cu(OH)_2$ (495 cm^{-1}), and Cu(III) oxides (606 cm^{-1}, could be $Cu(III)O_2^-$-like species)[6]. Cu(III) oxide was regarded as the active phase for OER catalysis[6], in line with the OER current observed in Supplementary Figure 4e. Source data are provided as a Source Data file.

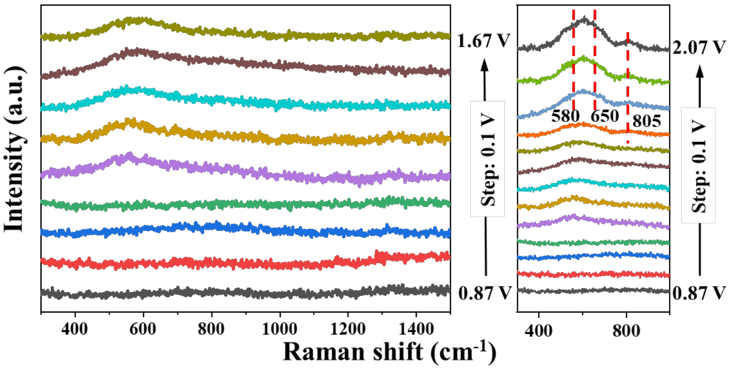

Supplementary Figure 10. In situ Raman spectra of Au foil in 0.1 M Fe-free KOH under applied potentials ranging from 0.87 V to 2.07 V vs RHE. In situ Raman showed a broad band centered around ca. 580 cm^{-1} and a shoulder at ca. 650 cm^{-1}. The former is ascribed to the Au–O vibration of gold surface oxide and the latter is assigned to Au-OH of hydroxides/oxyhydroxides[8,18,19] The characteristic Raman peaks indicate the presence of $Au(OH)_x$, AuOOH, and AuO_x on the surface of oxidized Au electrodes. Source data are provided as a Source Data file.

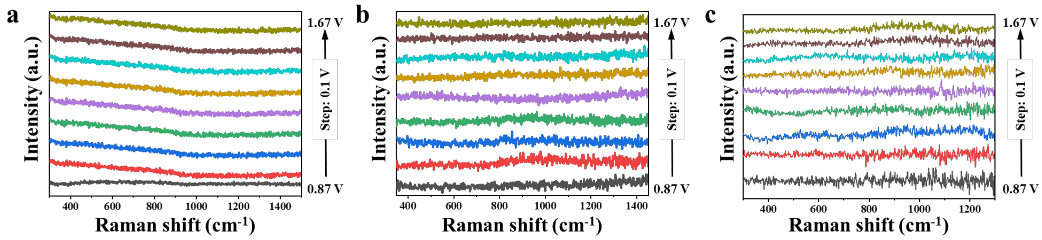

Supplementary Figure 11. In situ Raman spectra of metal foils. (a-c) Ti, Sn, Nb. Raman spectra under applied potentials ranging from 0.87 V to 1.67 V vs RHE. In situ Raman analysis exhibits no characteristic peaks. This could be ascribed to the formation of a passive layer of metal oxides/hydroxides on the surface. Here we take the Ti electrode as an example to interpret the formation of surface oxides/hydroxides. The anodic behavior of Ti in KOH has been investigated by Prof. Branko N. Popov in 2002[10]. They showed the formation of $Ti(OH)_3$ and $TiO_2{\cdot}H_2O$ on the surface, which could not be probed by Raman analysis due to the ultrathin nature. XPS was conducted to confirm the formation of surface oxides and hydroxides. It showed apparent lattice O and surface OH in the O *1s* spectra and cation peaks in the metal spectra (Supplementary Fig. 15). Similar passivation could occur on the surface of Nb and Sn electrodes[11,12], given the absence of redox peaks at the potential window here. Source data are provided as a Source Data file.

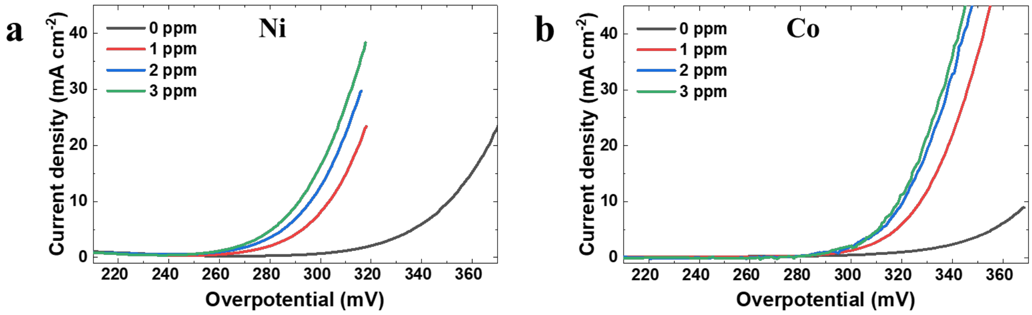

Supplementary Figure 12. Polarization curves of nickel and cobalt foils in KOH with different Fe concentrations. Backward scan; Scan rate: 1 mV s^{-1}; IR corrected. Source data are provided as a Source Data file.

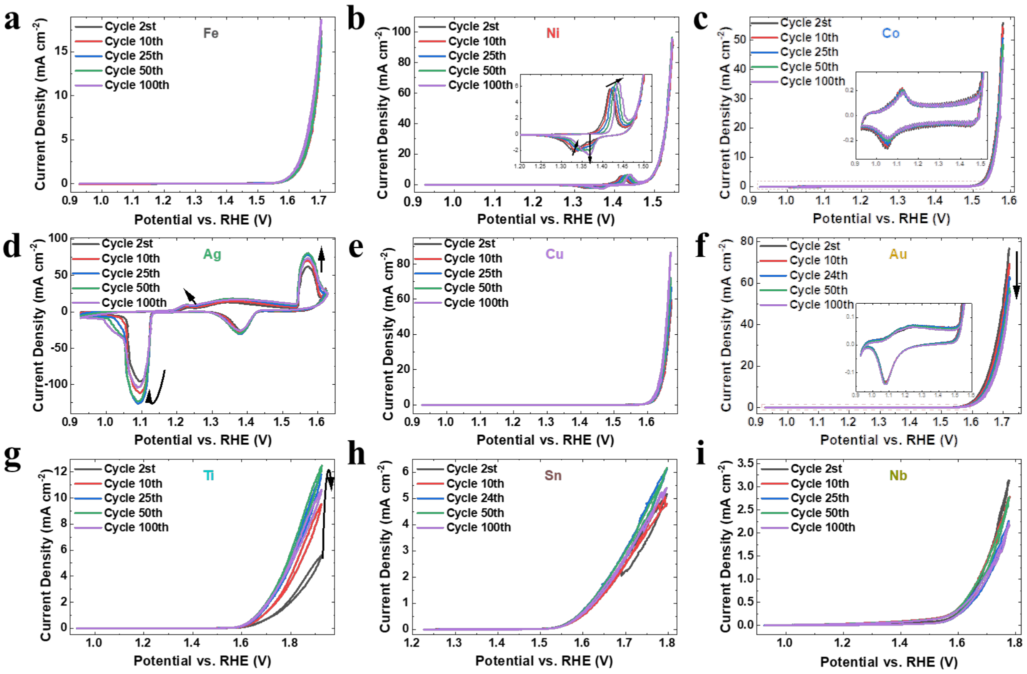

Supplementary Figure 13. 100 repetitive CV curves of metal foils in 1 M Fe-3ppm KOH at a scan rate of 20 mV s^{-1}. (a-i): Fe, Ni, Co, Ag, Cu, Au, Ti, Sn, Nb. Source data are provided as a Source Data file.

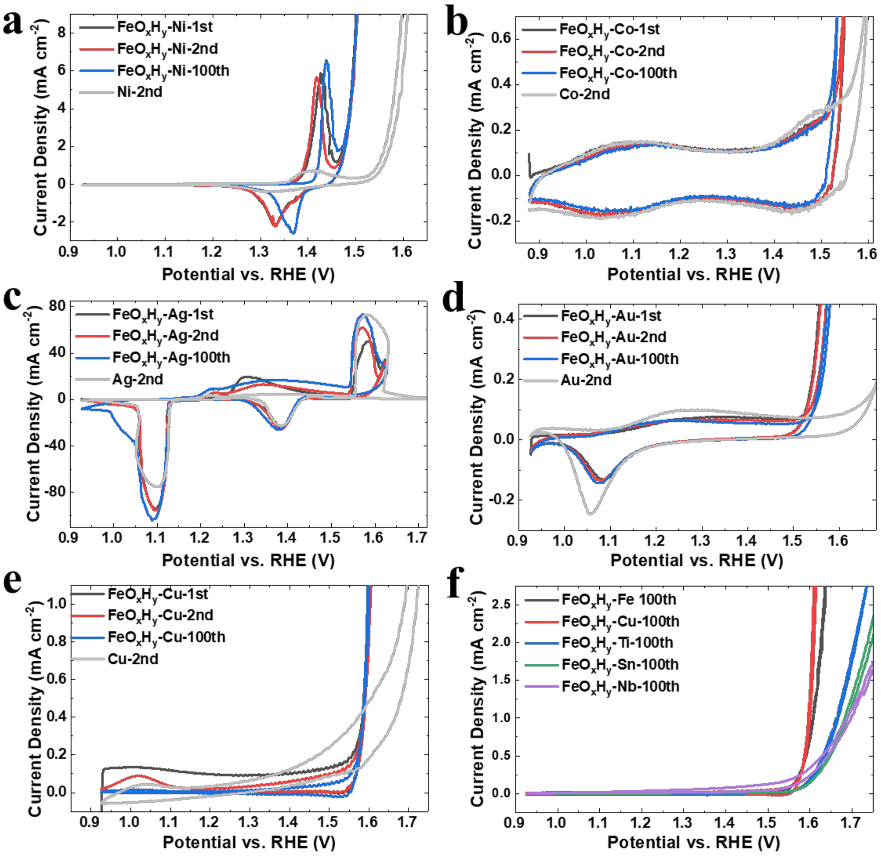

Supplementary Figure 14. Redox peaks in 1 M Fe-free and Fe-3ppm KOH at a scan rate of 20 mV s^{-1}. Source data are provided as a Source Data file.

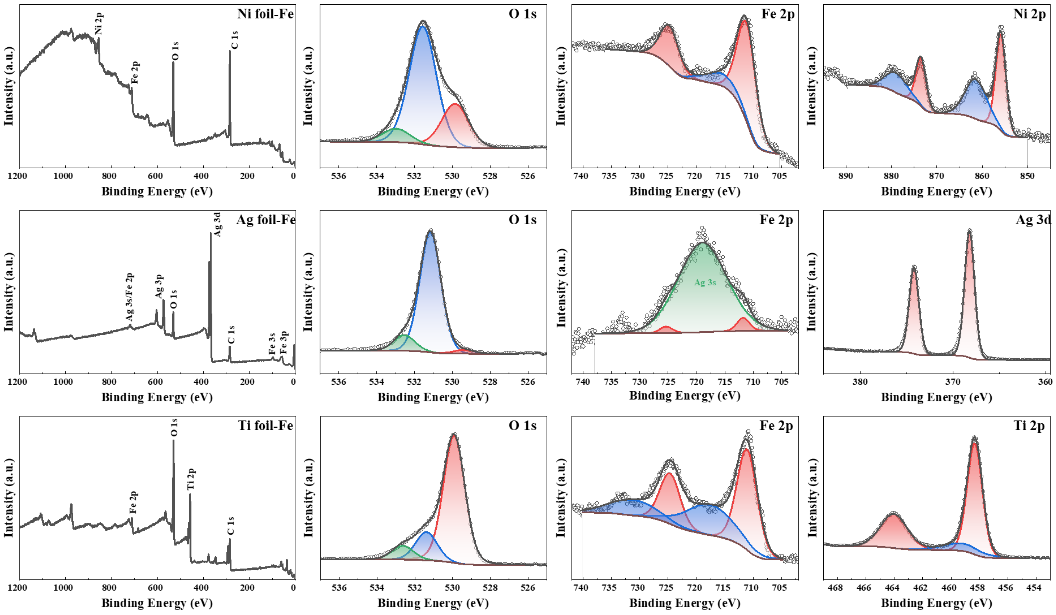

Supplementary Figure 15. XPS spectrum of nickel, silver, and titanium foils after OER test in 1 M Fe-3ppm KOH. Source data are provided as a Source Data file.

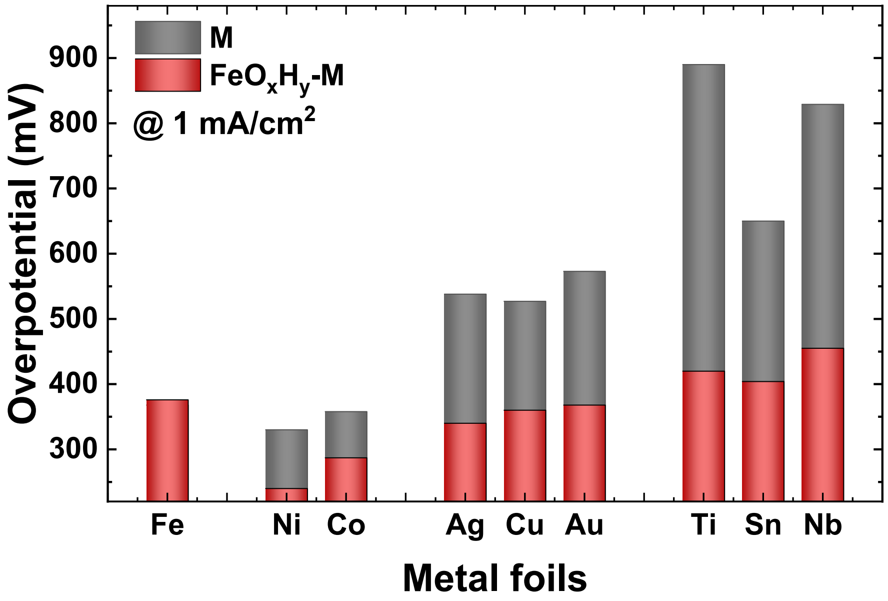

Supplementary Figure 16. Overpotentials required to achieve the current density of 1 mA cm^{-2}. Source data are provided as a Source Data file.

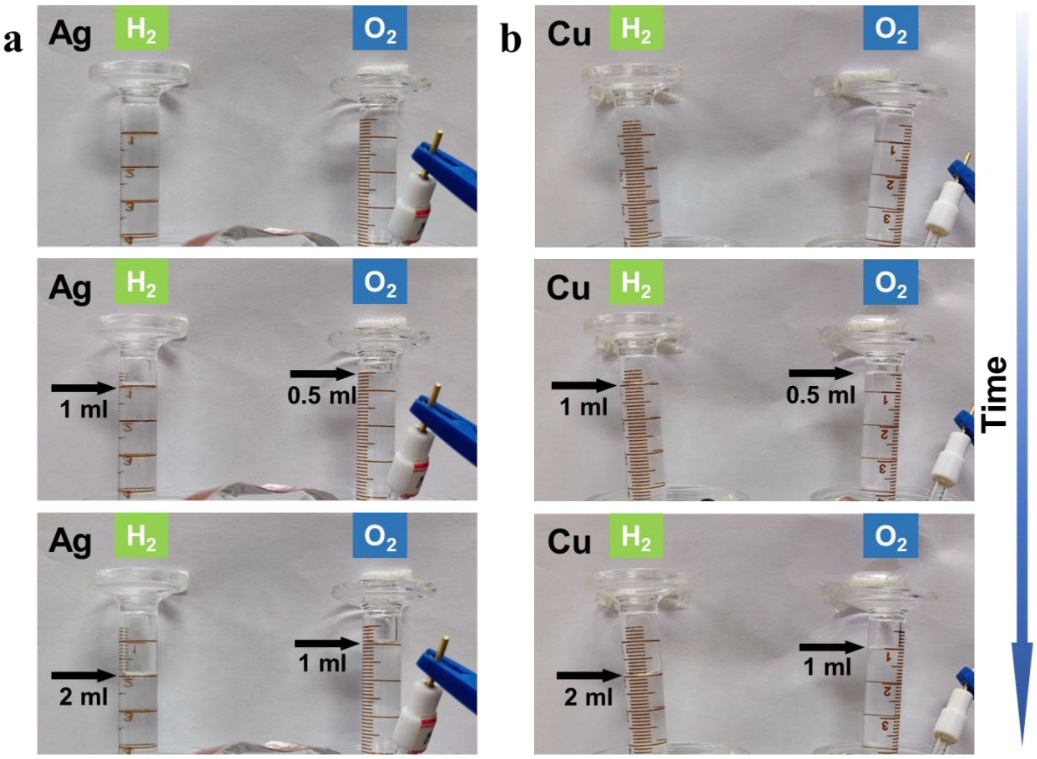

Supplementary Figure 17. Photographs of collected gases at the cathode (H_2) and anode (O_2) during water electrolysis. a) FeO_xH_y@Ag. b) FeO_xH_y@Cu. The Faradaic efficiencies have been probed by the drainage method (measured at the current density of 10 mA cm^{-2}). The H_2/O_2 volume ratio was roughly 2:1, which was close to the theoretical value. It suggests a nearly 100% faradaic efficiency for FeO_xH_y@Cu and FeO_xH_y@Ag. This indicates that most of the current goes to OER catalysis.

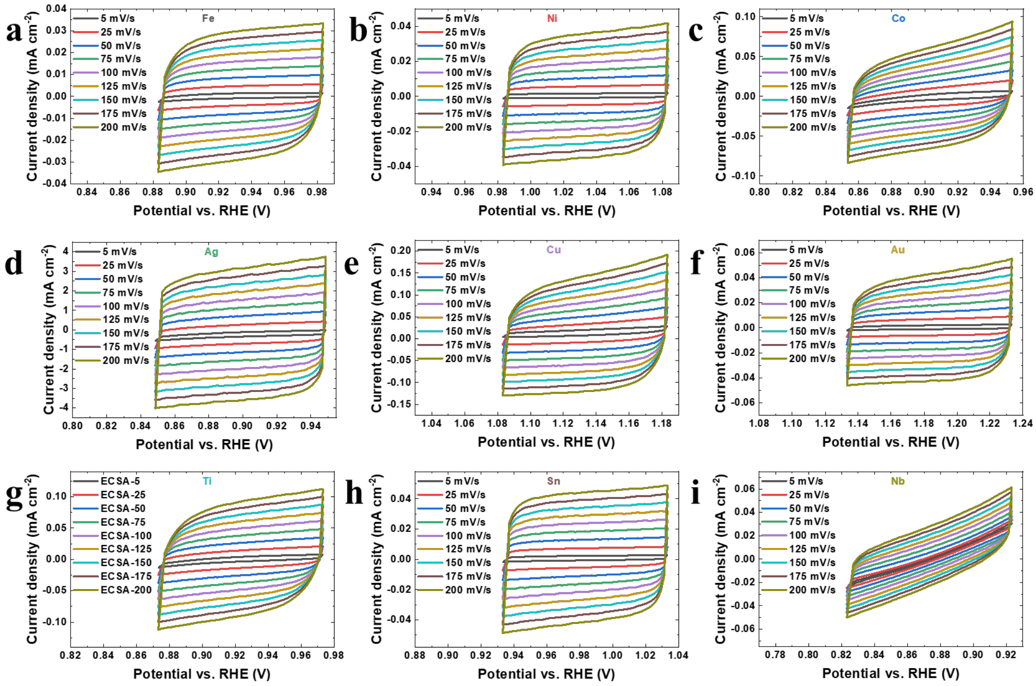

Supplementary Figure 18. Double-layer cyclic voltammograms of metal foils in 1 M Fe-3ppm KOH at scan rates ranging from 5 to 200 mV s^{-1}. (a-i): Fe, Ni, Co, Ag, Cu, Au, Ti, Sn, Nb. Source data are provided as a Source Data file.

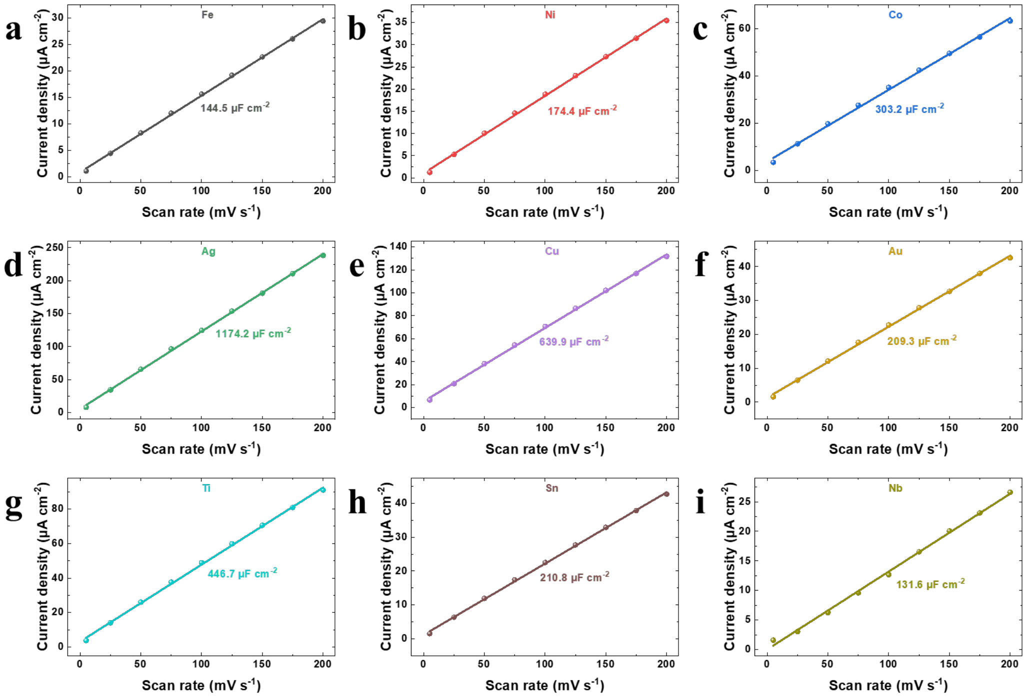

Supplementary Figure 19. Capacitive current densities versus scan rates of metal foils in 1 M Fe-3ppm KOH. (a-i): Fe, Ni, Co, Ag, Cu, Au, Ti, Sn, Nb. The linear slope is equivalent to the double-layer capacitance C_{dl}. Source data are provided as a Source Data file.

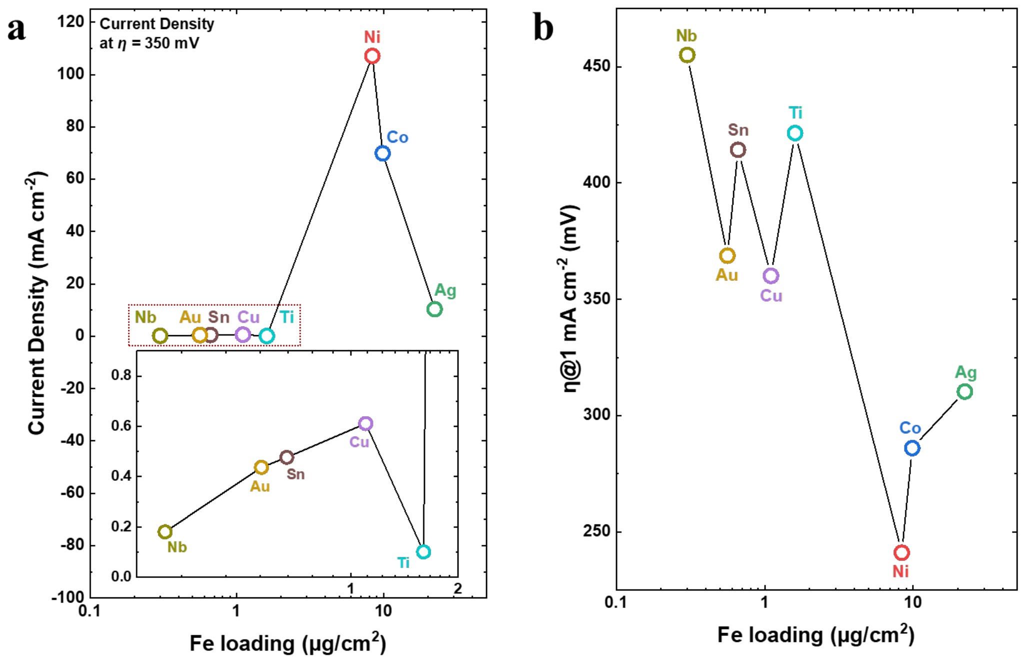

Supplementary Figure 20. OER catalytic performances plotting against iron loadings. (a) Current density at η=350 mV and (b) overpotential at 1 mA cm^{-2} versus iron loading. Source data are provided as a Source Data file.

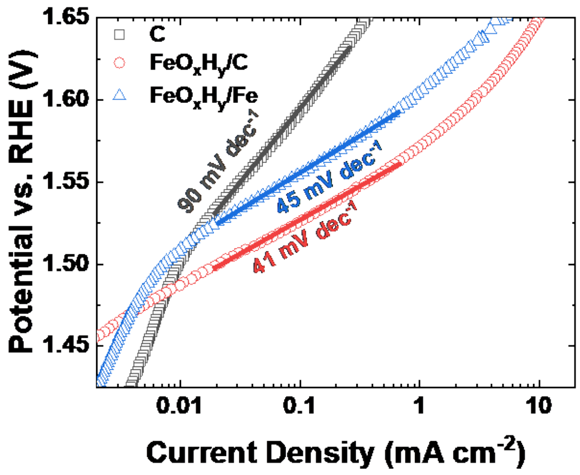

Supplementary Figure 21. Tafel slope of FeO_xH_y deposited on conductive carbon cloth. Source data are provided as a Source Data file.

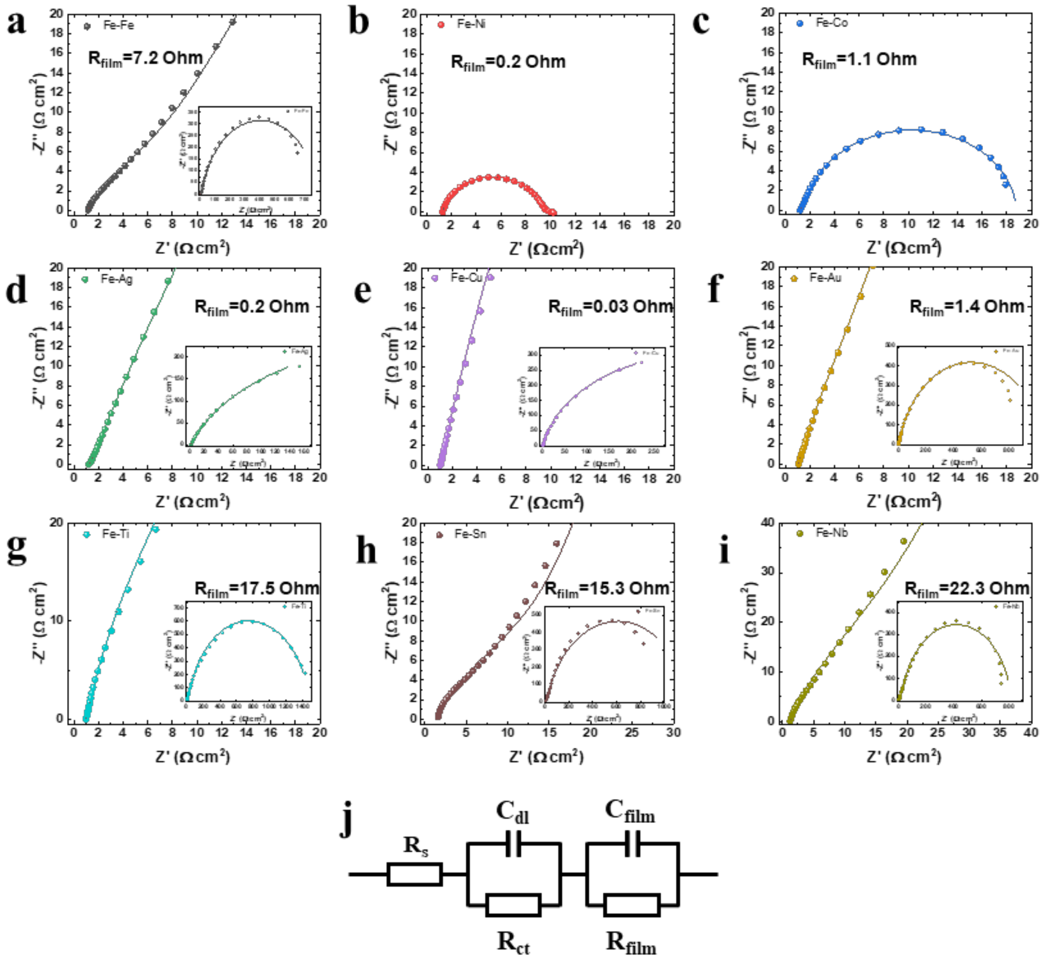

Supplementary Figure 22. Electrochemical impedance analysis. (a-i) Nyquist plots and the corresponding fitted curves of the metal foils of a) Fe, b) Ni, c) Co, d) Ag, e) Cu, f) Au, g) Ti, h) Sn, i) Nb in 1 M Fe-3ppm KOH, and j) the equivalent circuit model for EIS fitting. (b) The equivalent Viogot circuit used to model the OER[20]. There is only one semi-circle in the Nyquist plots of Ni, Co, Ag, Cu, and Au substrates, indicating the electron transport resistances are all negligible along the through-plane direction of the catalyst (for both FeOxHy and electrochemically formed oxyhydroxide). In this case, the potential difference of over 350 mV at 10 mA cm^{-2} between the most active one and the worst active one (corresponding to a 1-2 order of magnitude difference in catalytic activities, Fig. 1d) cannot be rationalized by the electron transfer resistances (given that there is not apparent resistance on these substrates). Therefore, we rule out the contribution of electron transport resistance to the catalytic activity difference of FeO_xH_y@Ni, Co, Ag, Cu, and Au substrates. For Fe, Ti, Sn, and Nb substrates we have observed two semi-circular responses in the Nyquist plots. It indicates that the electron transport could have a negative effect on their activities. The negative effect is reflected by the relatively larger Tafel slopes (~50-120 mV dec^{-1}) of FeO_xH_y@ Fe, Ti, Sn, and Nb substrates over that of FeO_xH_y@carbon cloths (~40 mV dec^{-1}, Supplementary Fig. 21). Source data are provided as a Source Data file.

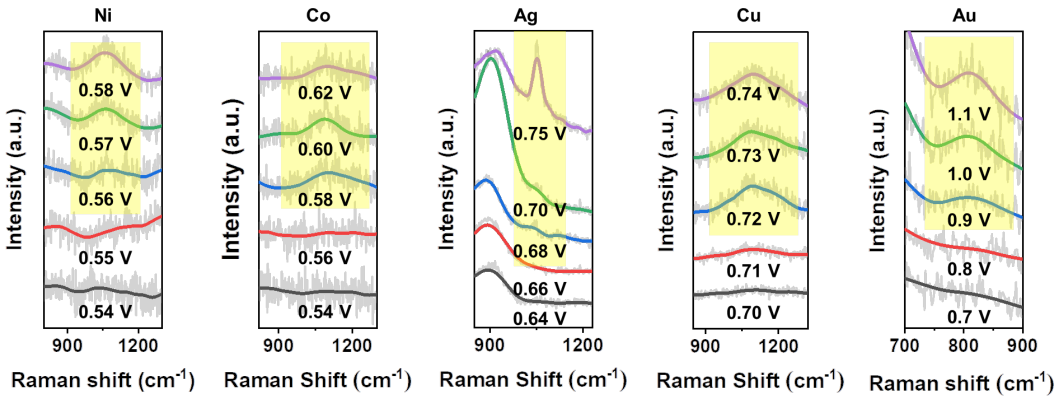

Supplementary Figure 23. In situ Raman spectra to probe active oxygen species. The electrolyte is 0.1 M Fe-free KOH. Source data are provided as a Source Data file.

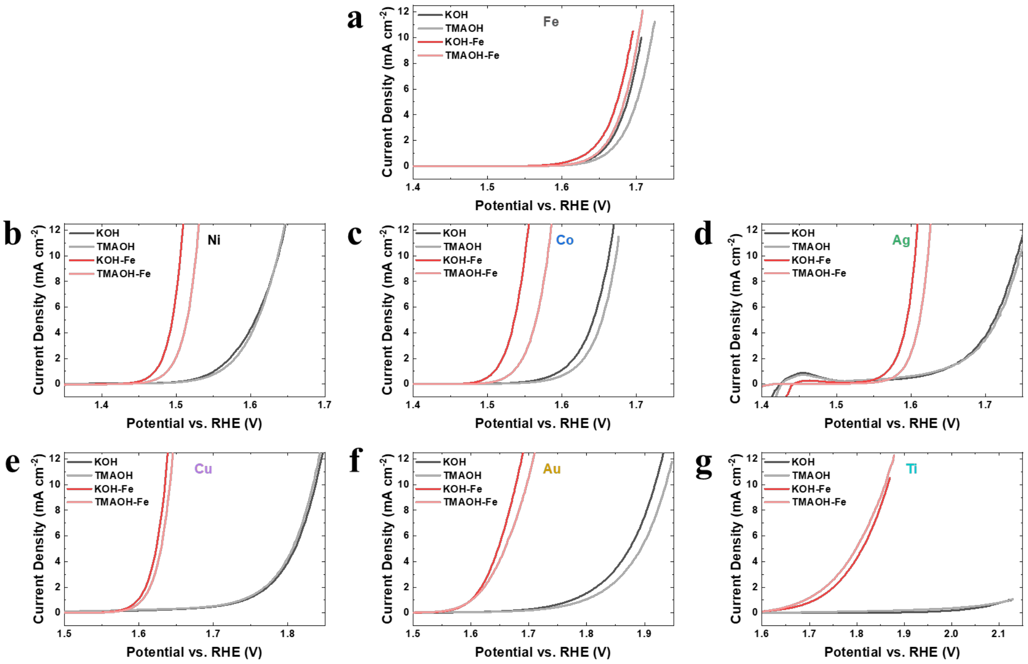

Supplementary Figure 24. Polarization curves of metal foils in 1 M KOH and 1 M TMAOH at a scan rate of 1 mV s^{-1}. (a-g) : Fe, Ni, Co, Ag, Cu, Au, Ti. Source data are provided as a Source Data file.

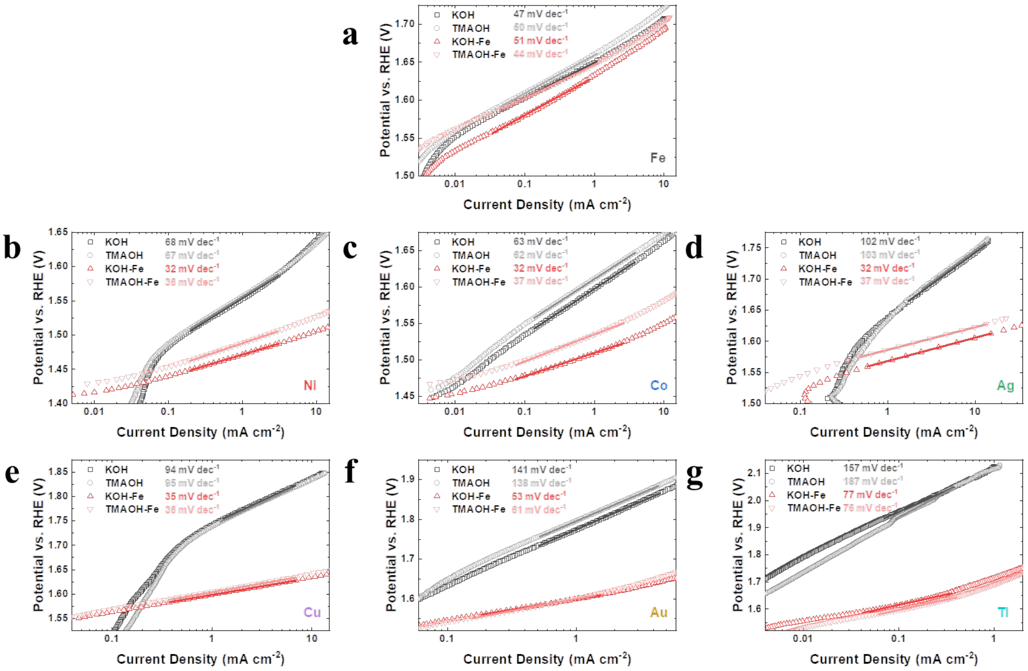

Supplementary Figure 25. Tafel plots of metal foils in 1 M KOH and 1 M TMAOH. (a-g): Fe, Ni, Co, Ag, Cu, Au, Ti. The Tafel plots are based on the backward CV curves (1 mV s^{-1}). Source data are provided as a Source Data file.

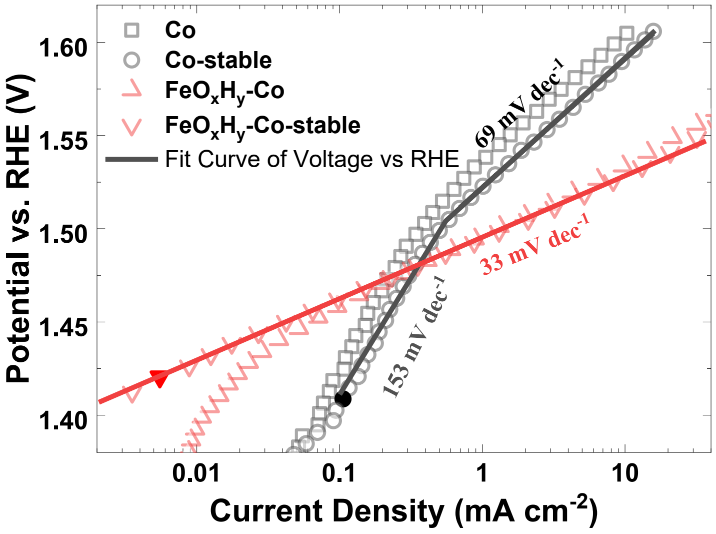

Supplementary Figure 26. Tafel plots of cobalt foam in Fe-free and Fe-sparking 1 M KOH. The Tafel plots are based on the backward CV curves (1 mV s^{-1}). Source data are provided as a Source Data file.

3. Supplementary Tables

Supplementary Table 1. Comparison of our work with previously reported ones.

Materials	Techniques	Active sites	Are active oxygen species the precursors of produced O_2	Conclusion	Ref.
FeM(O)OH	In situ Raman	Fe	No	The deposition of FeO_xH_y does not affect the presence of active oxygen species; Cooperative catalysis (strong synergy) is revealed between FeO_xH_y and active oxygen species, where proton transfer and/or diffusion play an essential role.	This work
FeNi(O)OH	Chemical probes	Ni	Yes	Fe induces the formation of active oxygen species; Fe modulates the interfacial interaction between active oxygen species and OH^- groups in the inner Helmholtz plane by modifying the interfacial proton diffusion.	[21]Alexis Grimaud
FeNi(O)OH	In situ Raman and isotope experiment	Fe	No	Fe incorporation does not affect the presence of active oxygen species; Stabilizing active oxygen species by larger-sized cations ($Cs^+ > K^+ > Na^+ > Li^+$) enhances catalytic activities of both Ni(O)OH and NiFe(O)OH; Fail to link the active oxygen species to the synergy effect between FeO_xH_y on Ni(O)OH	[22]Marc T. M. Koper
FeNi(O)OH	In situ Raman and isotope experiment	Fe	No	Fe incorporation prohibits the oxygen exchange of active oxygen species; The catalytic pathway shifts from a lattice oxygen mechanism in Ni(O)OH to an adsorbate evolution mechanism in NiFe(O)OH	[23]Xile Hu
FeNi(O)OH	In situ Raman and pH dependence analysis	Ni	Yes	Active oxygen species are the precursors of O_2 molecules produced by catalysis	[24]Wilson A. Smith

Supplementary Table 2. Fe loadings of all metal foils after OER test in 1 M Fe-3ppm KOH.

Metal foil	Fe	Ni	Co	Ag	Cu	Au	Ti	Sn	Nb
Fe Loading (μg cm^{-2})	-	8.38	9.86	22.28	1.10	0.56	1.60	0.66	0.30

Supplementary Table 3. Overpotentials at 10 mA cm^{-2} and Tafel slopes of metal foils after repetitive CV test in 1 M Fe-free KOH and 1 M Fe-3ppm KOH.[#]

	Overpotential at 10 mA cm^{-2} (mV)		Tafel slope (mV dec^{-1})		Active oxygen (Superoxo)
	Fe-free	Fe-3ppm	Fe-free	Fe-3ppm	Fe-free
Fe	454.5±20.5	452.0±2.8	47.6±2.1	48.0±2.6	/
Ni	382.5±6.4	280.5±4.9	59.3±7.8	32.3±0.6	√
Co	420.5±10.6	325.5±10.6	60.3±3.8	32.6±2.1	√
Ag	510.0±2.8	361.5±16.3	80.0±20.7	33.7±2.9	√
Cu	589.5±31.8	405.5±3.5	86.3±7.1	35.0±1.0	√
Au	674.5±29.0	428.5±6.4	129.0±14.4	51.0±3.5	√ *
Ti	890.5±4.9 @1 mA cm^{-2}	636.0±17.0	173.0±17.7	67.0±11.8	/
Sn	797.5±14.8 @1 mA cm^{-2}	>654.0	170.5±4.9	110±5.7	/
Nb	823.9±7.2 @1 mA cm^{-2}	>664.0	162.5±14.8	128±12.7	/

[#] Standard errors were calculated from the standard deviations from three measurements.

* Peroxide group (OOH).

Supplementary Table 4. Comparison of catalytic performances of metal foils in Fe-containing KOH solution in literature.#

	This work		[25]Shannon W. Boettcher		[7]Boon Siang Yeo	
	η at 1 mA cm^{-2} (mV)	Tafel slope (mV/dec)	η at 1 mA cm^{-2} (mV)	Tafel slope (mV/dec)	η at 1 mA cm^{-2} (mV)	Tafel slope (mV/dec)
Solution	3ppm Fe		1ppm Fe		0.3 mM Fe	
Fe	376	48.0±2.6		/	~428	42±3
Ni	240	32.3±0.6		/	~275	33±1
Co	287	32.6±2.1		/	~310	33±1
Ag	340	33.7±2.9		/	~338	40±2
Cu	360	35.0±1.0	~431	54	~366	29±0.3
Au	368	51.0±3.5	~340	49	~387	55±2
Ti	420	67.0±11.8		/	~1250	233±10
Sn	404	110±5.7		/		/
Nb	455	128±12.7		/		/

The trend of catalytic performances is similar in different literatures. The difference in values should be attributed to the variation in the activation process, Fe concentration, and electrochemical protocol to record the performances. Standard errors were calculated from the standard deviations from three measurements.

Supplementary Table 5. Comparison of TOFs of NiFe-based OER catalysts in literature.

Materials	TOF (s^{-1}) at η = 300mV	Active sites	Reference
FeO_xH_y-Ni	0.77	Fe site	This work
Ni−Fe films	~0.50	Fe site	[3]
F-NiFe-A	2.62 ± 0.28	Fe site	[26]
NiFe-LDH HMS	~0.86	Fe site	[27]
Fe^{2+}-NiFe LDH	~0.18	Fe site	[28]
NiFeOOH/Au	~0.45	Fe site	[29]
$Fe(PO_3)_2/Ni_2P$	0.12	Fe site	[30]
$Ni_{45}Fe_{55}O_xH_y$	~0.26	Fe site	[31]
Ultrathin NiFe LDH	0.39	Fe site	[32]

Supplementary Table 6. Comparison of TOFs of CoFe-based OER catalysts in literature.

Materials	TOF (s^{-1}) at η = 350mV	Active sites	Reference
FeO_xH_y-Co	1.03	Fe site	This work
$Co_{1-x}Fe_x(OOH)$	0.8 ± 0.3	Fe site	33
α-$Co_{0.9}Fe_{0.1}(OH)_x$	~1.87	Fe site	34
β-$Co_{0.9}Fe_{0.1}(OH)_x$	~0.26	Fe site	
Fe_1Co_1-ONS	~0.04	Fe site	35
Co_2Fe_1 LDH	~0.22	Fe site	36
$V_{0.2}$-CoFe-LDH	~0.76	Fe site	37

Supplementary Table 7. Fitting results of Nyquist plots of metal foils in 1 M Fe-3ppm KOH.

Materials	R_s $\Omega\cdot cm^2$	C_{dl} mF/cm^2	R_{ct} $\Omega\cdot cm^2$	C_{film} mF/cm^2	R_{film} $\Omega\cdot cm^2$
Fe	1.223	0.5879	788.5	1.295	7.249
Ni	1.288	2.057	7.88	64.18	0.182
Co	1.197	5.821	16.48	48.00	1.105
Ag	1.180	5.380	615.2	141.0	0.205
Cu	1.008	3.516	720.4	10.35	0.029
Au	1.082	0.8575	1053	3.114	1.350
Ti	0.9725	0.1556	1459	0.2713	17.45
Sn	1.338	0.3111	1190	0.4102	15.28
Nb	1.204	0.2385	796.4	0.4447	22.34

Supplementary References

1. Lyons, M. E. G. & Brandon, M. P. A comparative study of the oxygen evolution reaction on oxidised nickel, cobalt and iron electrodes in base. *J. Electroanal. Chem.* **641**, 119–130 (2010).
2. Larramona, G. & Gutiérrez, C. The Passive Film on Iron at pH 1–14: A Potential-Modulated Reflectance Study. *J. Electrochem. Soc.* **136**, 2171–2178 (1989).
3. Louie, M. W. & Bell, A. T. An Investigation of Thin-Film Ni−Fe Oxide Catalysts for the Electrochemical Evolution of Oxygen. *J. Am. Chem. Soc.* **135**, 12329−12337 (2013).
4. Desilvestro, J., Corrigan, D. A. & Weaver, M. J. Characterization of Redox States of Nickel Hydroxide Film Electrodes by In Situ Surface Raman Spectroscopy. *J. Electrochem. Soc.* **135**, 885–892 (1988).
5. Iwasaki, N., Sasaki, Y. & Nishina, Y. Ag electrode reaction in NaOH solution studied by in-situ Raman spectroscopy. *Surf. Sci.* **198**, 524–540 (1988).
6. Deng, Y., Handoko, A. D., Du, Y., Xi, S. & Yeo, B. S. In Situ Raman Spectroscopy of Copper and Copper Oxide Surfaces during Electrochemical Oxygen Evolution Reaction: Identification of CuIII Oxides as Catalytically Active Species. *ACS Catal.* **6**, 2473–2481 (2016).
7. Gong, L., Koh, J. & Yeo, B. S. Mechanistic Study of the Synergy between Iron and Transition Metals for the Catalysis of the Oxygen Evolution Reaction. *ChemSusChem* **11**, 3790–3795 (2018).
8. Diaz-Morales, O., Calle-Vallejo, F., Munck, C. de & Koper, M. T. M. Electrochemical water splitting by gold: evidence for an oxide decomposition mechanism. *Chem. Sci.* **4**, 2334–2343 (2013).
9. Doyle, R. L. & Lyons, M. E. G. The mechanism of oxygen evolution at superactivated gold

electrodes in aqueous alkaline solution. *J. Solid State Electrochem.* **18**, 3271–3286 (2014).

10. Prusi, A., Arsov, Lj., Haran, B. & Popov, B. N. Anodic Behavior of Ti in KOH Solutions. *J. Electrochem. Soc.* **149**, B491-B498 (2002).
11. Huang, B. X., Tornatore, P. & Li, Y.-S. IR and Raman spectroelectrochemical studies of corrosion films on tin. *Electrochim. Acta* **46**, 671–679 (2001).
12. Arsova, I., Prusi, A., Grcev, T. & Arsov, L. Electrochemical characterization of the passive films formed on niobium surfaces in H2SO4 solutions. *J. Serbian Chem. Soc.* **71**, 177–187 (2006).
13. Friebel, D. *et al.* Identification of Highly Active Fe Sites in (Ni,Fe)OOH for Electrocatalytic Water Splitting. *J. Am. Chem. Soc.* **137**, 1305–1313 (2015).
14. Yeo, B. S. & Bell, A. T. Enhanced Activity of Gold-Supported Cobalt Oxide for the Electrochemical Evolution of Oxygen. *J. Am. Chem. Soc.* **133**, 5587–5593 (2011).
15. Moysiadou, A., Lee, S., Hsu, C.-S., Chen, H. M. & Hu, X. Mechanism of Oxygen Evolution Catalyzed by Cobalt Oxyhydroxide: Cobalt Superoxide Species as a Key Intermediate and Dioxygen Release as a Rate-Determining Step. *J. Am. Chem. Soc.* **142**, 11901–11914 (2020).
16. Waterhouse, G. I. N., Bowmaker, G. A. & Metson, J. B. The thermal decomposition of silver (I, III) oxide: A combined XRD, FT-IR and Raman spectroscopic study. *Phys. Chem. Chem. Phys.* **3**, 3838–3845 (2001).
17. Savinova, E. R. *et al.* On the mechanism of Ag(111) sub-monolayer oxidation: a combined electrochemical, in situ SERS and ex situ XPS study. *Electrochim. Acta* **46**, 175–183 (2000).
18. Yeo, B. S., Klaus, S. L., Ross, P. N., Mathies, R. A. & Bell, A. T. Identification of Hydroperoxy Species as Reaction Intermediates in the Electrochemical Evolution of Oxygen on Gold. *ChemPhysChem* **11**, 1854–1857 (2010).

19. Desilvestro, J. & Weaver, M. J. Surface structural changes during oxidation of gold electrodes in aqueous media as detected using surface-enhanced Raman spectroscopy. *J. Electroanal. Chem. Interfacial Electrochem.* **209**, 377–386 (1986).

20. Morales-Guio, C. G., Liardet, L. & Hu, X. Oxidatively Electrodeposited Thin-Film Transition Metal (Oxy)hydroxides as Oxygen Evolution Catalysts. *J. Am. Chem. Soc.* **138**, 8946–8957 (2016).

21. Yang, C., Fontaine, O., Tarascon, J.-M. & Grimaud, A. Chemical Recognition of Active Oxygen Species on the Surface of Oxygen Evolution Reaction Electrocatalysts. *Angew. Chem. Int. Ed.* **56**, 8652–8656 (2017).

22. Garcia, A. C., Touzalin, T., Nieuwland, C., Perini, N. & Koper, M. T. M. Enhancement of Oxygen Evolution Activity of Nickel Oxyhydroxide by Electrolyte Alkali Cations. *Angew. Chem. Int. Ed.* **58**, 12999–13003 (2019).

23. Lee, S., Banjac, K., Lingenfelder, M. & Hu, X. Oxygen Isotope Labeling Experiments Reveal Different Reaction Sites for the Oxygen Evolution Reaction on Nickel and Nickel Iron Oxides. *Angew. Chem.* **131**, 10401–10405 (2019).

24. Trześniewski, B. J. *et al.* In Situ Observation of Active Oxygen Species in Fe-Containing Ni-Based Oxygen Evolution Catalysts: The Effect of pH on Electrochemical Activity. *J. Am. Chem. Soc.* **137**, 15112–15121 (2015).

25. Enman, L. J., Vise, A. E., Burke Stevens, M. & Boettcher, S. W. Effects of Metal Electrode Support on the Catalytic Activity of Fe(oxy)hydroxide for the Oxygen Evolution Reaction in Alkaline Media. *ChemPhysChem* **20**, 3089–3095 (2019).

26. Xu, Q. *et al.* Fluorination-enabled Reconstruction of NiFe Electrocatalysts for Efficient Water

Oxidation. *Nano Lett.* **21**, 492–499 (2021).

27. Zhang, C. *et al.* Hierarchical NiFe Layered Double Hydroxide Hollow Microspheres with Highly-Efficient Behavior toward Oxygen Evolution Reaction. *ACS Appl. Mater. Interfaces* **8**, 33697–33703 (2016).
28. Cai, Z. *et al.* Introducing Fe2+ into Nickel-Iron Layered Double Hydroxide: Local Structure Modulated Water Oxidation Activity. *Angew. Chem.* **130**, 9536–9540 (2018).
29. Chakthranont, P. *et al.* Effects of Gold Substrates on the Intrinsic and Extrinsic Activity of High-Loading Nickel-Based Oxyhydroxide Oxygen Evolution Catalysts. *ACS Catal.* **7**, 5399–5409 (2017).
30. Zhou, H. *et al.* Highly active catalyst derived from a 3D foam of $Fe(PO_3)_2/Ni_2P$ for extremely efficient water oxidation. *Proc. Natl. Acad. Sci. U.S.A.* **114**, 5607–5611 (2017).
31. Görlin, M. *et al.* Oxygen Evolution Reaction Dynamics, Faradaic Charge Efficiency, and the Active Metal Redox States of Ni–Fe Oxide Water Splitting Electrocatalysts. *J. Am. Chem. Soc.* **138**, 5603–5614 (2016).
32. Kuai, C. *et al.* Fully Oxidized Ni–Fe Layered Double Hydroxide with 100% Exposed Active Sites for Catalyzing Oxygen Evolution Reaction. *ACS Catal.* **9**, 6027–6032 (2019).
33. Burke, M. S., Kast, M. G., Trotochaud, L., Smith, A. M. & Boettcher, S. W. Cobalt–Iron (Oxy)hydroxide Oxygen Evolution Electrocatalysts: The Role of Structure and Composition on Activity, Stability, and Mechanism. *J. Am. Chem. Soc.* **137**, 3638–3648 (2015).
34. Liu, Y. *et al.* Electroactive Edge-Site-Enriched α-Co 0.9Fe0.1(OH)x Nanoplates for Efficient Overall Water Splitting. *ChemElectroChem* **6**, 2415–2422 (2019).
35. Zhuang, L. *et al.* Ultrathin Iron-Cobalt Oxide Nanosheets with Abundant Oxygen Vacancies for

the Oxygen Evolution Reaction. *Adv. Mater.* **29**, 1606793 (2017).

36. Feng, L. *et al.* A Highly Active CoFe Layered Double Hydroxide for Water Splitting. *ChemPlusChem* **82**, 483–488 (2017).

37. Singh, B. & Indra, A. Tuning the properties of CoFe-layered double hydroxide by vanadium substitution for improved water splitting activity. *Dalton Trans.* **50**, 2359–2363 (2021).
